# Variations of Essential Oil Constituents in Oregano (*Origanum vulgare* subsp. *viridulum* (= *O. heracleoticum*) over Cultivation Cycles

**DOI:** 10.3390/plants9091174

**Published:** 2020-09-10

**Authors:** Edoardo Napoli, Antonio Giovino, Alessandra Carrubba, Vandana How Yuen Siong, Carmelo Rinoldo, Onofrio Nina, Giuseppe Ruberto

**Affiliations:** 1 Istituto del CNR di Chimica Biomolecolare, Via P. Gaifami 18, 95126 Catania, Italy; edoardo.napoli@icb.cnr.it (E.N.); vale.marte.92@hotmail.it (V.H.Y.S.); giuseppe.ruberto@icb.cnr.it (G.R.); 2 Consiglio per la Ricerca in Agricoltura e l’Analisi dell’Economia Agraria—Centro di Ricerca Difesa e Certificazione (CREA-DC), Strada Statale 113 km 245.500, 90011 Bagheria, Italy; antonio.giovino@crea.gov.it; 3 Dipartimento di Scienze Agrarie, Alimentari e Forestali—Università di Palermo, Viale delle Scienze Ed. 4 Ingr. L, 90128 Palermo, Italy; 4 Assessorato Regionale dell’Agricoltura, dello Sviluppo Rurale e della Pesca Mediterranea, Ispettorato dell’Agricoltura di Agrigento—U.O. S6.11, U.I.A. di Aragona, Via Scalo Caldare, 92021 Aragona, Italy; carmelo.rinoldo@regione.sicilia.it (C.R.); onofrio.nina@regione.sicilia.it (O.N.)

**Keywords:** oregano, essential oil, aromatic plants cultivation, Sicily, active metabolites, thymol, harvest time

## Abstract

Oregano is—probably—the most appreciated and widespread aromatic plant in Sicily. With the aim of evaluating the modifications of oregano’s essential oil composition over time, between 2013 and 2015 six weekly samplings of three different oregano plantations were carried out, from the beginning of flowering (early May) until the traditional harvest moment (end of June). Samples were hydrodistilled and the obtained essential oils (EOs) were evaluated by means of a combination of GC–FID and GC–MS. The *Origanum* plants under study were demonstrated to belong to the high-yielding, thymol-type biotypes, with thymol, γ-terpinene and *p*-cymene as three main components, among the total of about 50 of the evaluated EOs. In each location, EO yields were found to increase throughout survey dates. Significant variations were found in many EO components, both across years and throughout harvest dates within locations. The choice of the harvest moment was confirmed to be crucial in assessing quality aspects of oregano.

## 1. Introduction

The genus *Origanum* (*Lamiaceae*) contains about 70 taxa, including species, subspecies and hybrids native to and widespread in the entire Mediterranean region [1,2,3]. According to Bartolucci et al. [4] and Govaerts et al. [5], *Origanum vulgare* subsp. *viridulum* (Martrin-Donos) Nyman (= *Origanum heracleoticum* L.; *O. vulgare* subsp. *viride* (Boissier) Hayek *sensu* Ietswaart (1980)) is the most common species growing in Sicily, yet scarce information is available about the phytochemical characteristics of this taxon, whereas many papers have been presented toward the study of *Origanum vulgare* subsp. *hirtum* (Link) Ietswaart, also known as “Greek oregano.” Ietswaart [1] discriminates *Origanum vulgare* subsp. *hirtum* from *O. vulgare* subsp. *viride* based on small morphological traits, the most important being the density of sessile glands on the abaxial surface of leaves, on bracts and on calices, reaching about up to 2000 per cm^2^ in the subsp. *hirtum* and up to 900 per cm^2^ in the subsp. *viride*. Since in oregano these sessile glands are the sites of synthesis and storage of the essential oils (EOs), their different numbers would explain the contrasting EO yields of these two taxa: the subsp. *viride* comprises individuals and populations with an EO content <2%, whereas subsp. *hirtum* comprises high-yielding (>2%) individuals [6]. Furthermore, a geographical distinction has been found as well; the low-yielding genotypes are mostly distributed in the northern (cooler) European areas, and the high-yielding genotypes are in the Southern (warmer) areas [6]. However, it is unquestionable that the high frequency of intraspecific hybridization leads to the presence of many populations with intermediate characters, and this outstanding morphological and chemical variability makes the classification of *Origanum* subspecies rather uneasy [7]. Sicilian oregano is up to 1 m tall, with white flowers in ovoidal spikes, and it is strongly aromatic in all its parts, due to the presence of many essential oil-bearing sessile glands on leaves and bracts. Due to its strong aromatic properties, this plant is traditionally used in Sicily for condiment purposes, and represents a basic ingredient for many traditional recipes [8,9]. Traditionally it was gathered from wild populations, but in recent years, increasing interest has been addressed to its specialized cultivation [10,11,12]. Indeed, oregano plays an increasingly important role as an industrial raw matter: both the entire plant and its biochemical derivatives, such as extracts and essential oils, are largely used in the food industry as a spice [13], as preservatives in meat storage [14,15] and as food products [16]. They also find use in pharmaceutical industry, because of their numerous well-known pharmacological properties [17,18,19,20,21,22,23,24,25,26,27].

Many studies have been conducted on oregano’s essential oils, exploring a large deal of its intraspecific variability. The occurrence of three well defined chemo-groups is generally acknowledged, namely: (a) the linalool, terpinen-4-ol and sabinene hydrate group; (b) the carvacrol and/or thymol group; (c) the sesquiterpenes group [6,28]. Since separated biosynthetic routes seem responsible for the in-plant production of these major chemical groups, it is reasonably deduced that the prevalence of one of them (hence the outcoming chemotype) is basically genetically determined [29,30]. Inside each chemotype, significant variations may occur, leading to a wide variability in the measured contents of certain chemical constituents. Many factors may play roles in these variations, and therefore in assessing the final chemical characteristics of *Origanum* essential oils. This field of study is huge, and much research has been conducted worldwide to explore the effects exerted on EO yield and quality by a number of environmental factors, such as altitude [31], temperature [30], harvest season and geographical position [32]. It is well recognized that cropping technique is also a crucial factor in assessing, within each chemotype, important variations of EO components [33,34].

Among the technical choices responsible for these modifications, harvest time surely has crucial importance. The existence of a well-defined development stage when the concentration of “active compounds” is the highest is well known to herbalists [35], and significant modifications in the EO yield and composition over time according to plant ontogeny have been assessed in many species [34], also including a number of *Labiatae* [36,37,38,39]. Most of them, including *Origanum*, are generally thought to achieve their best quality features when harvested at flowering time. In *O. vulgare* ssp. *hirtum*, significant modifications in yield and composition of the EO have been detected between plants collected in summer and in autumn, those from summer collection (i.e.**,** at full blooming) being endowed with higher EO content, higher amounts of **γ**-terpinene and lower amounts of *p*-cymene [32]. Significant variations may occur also within the flowering period; the progression in blooming stages may be also responsible for variations of EO quantity and composition. In a carvacrol-rich *Origanum* biotype, Król et al. [40] found increased EO content from the beginning to the end of flowering, and the obtained EOs were characterized by increasing amounts of thymol and decreasing *p*-cymene, **γ**-terpinene and carvacrol. Hence, the proper choice of harvest time may be crucial in assessing quality characteristics of the herbal product. Especially in semi-arid climates, where late spring and summer are characterized by strong environmental aridity, a delay or an anticipation of harvest means that plants are, or are not, exposed to very high temperatures and water shortage, ultimately affecting quality [41].

Hence, there is room for deep research about oregano crop potential in Sicily, with the aim of evaluating its morphological and quality traits over time, for deeper agro-industrial exploitation, both as a herbal product and as industrial raw matter.

**Figure 1:**
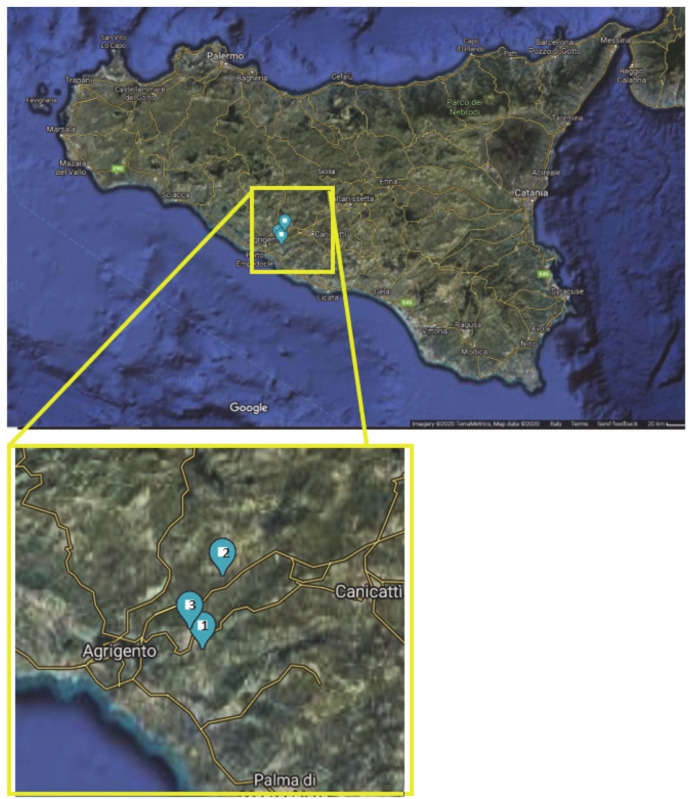
Geographical positions of the three studied *Origanum* fields. 1: Favara (AG), 37°18′38.8" N, 13°40′26.1″ E; 2: Racalmuto (AG), 37°22′15.5″ N, 13°41′40.7″ E; 3: Agrigento (AG), 37°19′37.1″ N, 13°39′35.2″ E.

This study reports the results obtained from a 3-year cultivation trial (from 2013 to 2015) of one *Origanum vulgare* subsp. *viridulum* (= *O. heracleoticum*) genotype in the southern part of Sicily (Figure 1), aimed at evaluating the variation in time of the yield and the aromatic profile of the obtained essential oils.

## 2. Results

**Figure 2:**
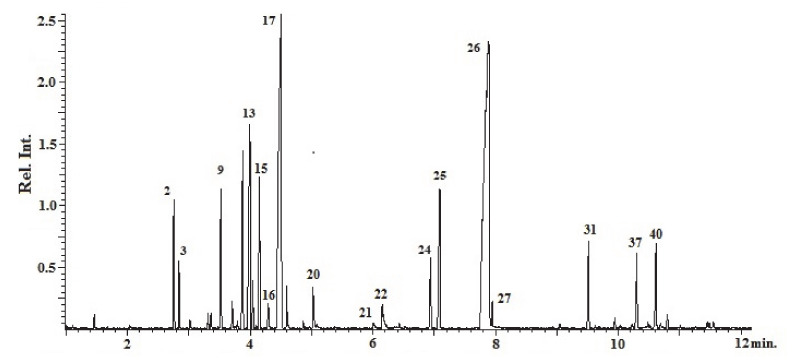
An example chromatogram of the EO from *Origanum heracleoticum* grown in Sicily. Peaks numbers correspond to the compounds listed in Table 1.

The analyses carried out in all three trial years (Table 1, Table 2 and Table 3, Figure 2) allowed for full characterization of the extracted EOs. More than 50 EO components were fully identified (50 compounds in 2013, 58 in 2014 and 60 in 2015), and for an easier comparison of the oils they were grouped into four classes: monoterpene hydrocarbons (also known as “monoterpenes”), oxygenated monoterpenes (monoterpenoids), sesquiterpenes and others. Monoterpenes, both hydrocarbons and oxygenated, represented more than 80% (84.26% to 96.82%) of the total compounds detected. Inside monoterpenes, hydrocarbons ranged between 19.71% and 47.61%, whereas oxygenated monoterpenes (i.e., monoterpenoids) showed a range of 45.27–65.47%. Total sesquiterpenes were always less represented (2.70–13.31%), and therefore no distinction was made between hydrocarbons (sesquiterpenes *sensu stricto*) and oxygenated forms (also known as “sesquiterpenoids”). The other compounds always reached amounts lower than 1%. Thymol (36.91–60.14%), γ-terpinene (11.59–24.14%) and *p*-cymene (2.56–9.38%) were the major components in all EOs.

**Figure 3:**
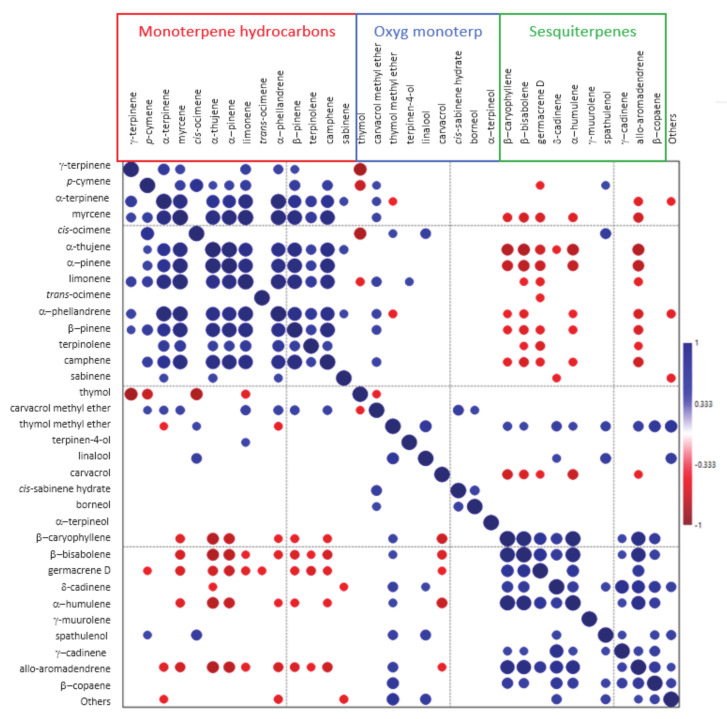
Correlation (Pearson’s *r*) plot of the major compounds detected in EO from *Origanum heracleoticum* cultivated in Sicily from 2013 to 2015 in 3 locations (*n* = 54). Positive and negative correlations are displayed in blue and red, respectively. Size and color intensity are proportional to the correlation coefficients. *r* values having significance values *p* > 0.05 were left blank.

Correlation analysis among the EO components (Figure 3) shows that monoterpene hydrocarbons are all positively and directly correlated among themselves.

The same, although to a slightly lesser extent, may be seen for sesquiterpenes. Otherwise, monoterpene hydrocarbons and sesquiterpenes are generally inversely correlated. Oxygenated monoterpenes show weak correlations both among themselves and with the compounds belonging to the other groups. Interesting exceptions are represented by thymol, inversely correlated with γ-terpinene (*r* = −0.816), *p*-cymene (*r* = −0.673) and *cis*-ocimene (*r* = −0.774); and linalool, positively correlated with *cis*-ocimene (*r* = 0.458).

The relationships between thymol, carvacrol, *p*-cymene and γ-terpinene, linked by their belonging to the same biosynthetic pathway (the “cymyl”-pathway [12,29]), are examined in Table 4. A high inverse correlation shows up between the sum thymol+carvacrol and *p*-cymene + γ-terpinene (*r* = −0.877; *p* < 0.001), and a positive correlation between thymol and carvacrol (*r* = 0.323; *p* = 0.017).

Thermal sums (°C) were positively associated (*r* = 0.252; *p* = 0.066) with the total amount of thymol + carvacrol + p-cymene + γ-terpinene; none of these compounds alone expressed a significant association with thermal sums, exception made for *p*-cymene (*r* = 0.287; *p*= 0.035). Finally, the content in thymol, carvacrol, their sum (thy+carv) and the sum of all four metabolites (thymol, carvacrol, *p*-cymene and γ-terpinene) were always inversely correlated with total rainfall amounts until harvest time.

The ANOVA performed on the EO yields and the detected amounts of the major EO components (Table 5) showed in all cases a strong influence of the “year” factor. The factor “location” expressed a significant influence on two compounds only (sabinene and *trans*-ocimene). These compounds, and carvacrol and borneol, were also influenced by the interaction “year x location.” A strong effect of the survey date within each location was evidenced in EO yield, in a number of compounds (α-thujene, α-pinene, camphene, β-pinene, myrcene, α-phellandrene, limonene, trans-ocimene, terpinolene, *cis*-sabinene hydrate, carvacrol methyl ether) and in total monoterpenes content (including monoterpene hydrocarbons + oxygenated monoterpenes).

**Figure 4:**
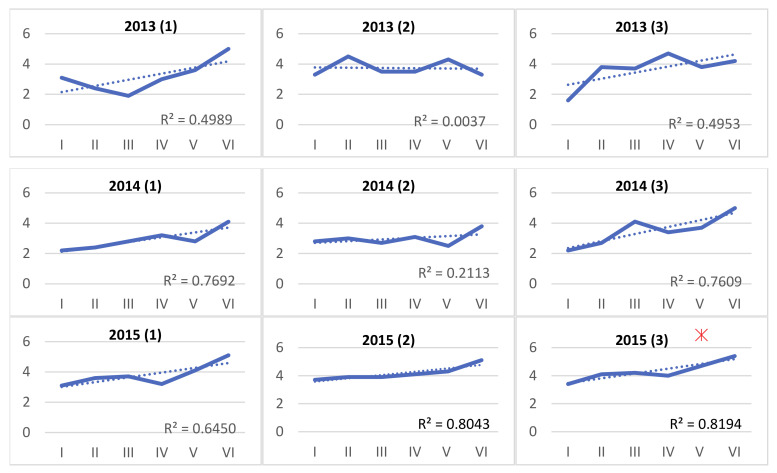
Trend over time of OE yield in *Origanum heracleoticum* in 3 years (2013 to 2015) and 3 locations (1 to 3). In each graph, regression lines (dotted lines) and corresponding R^2^ values are indicated. A red asterisk (*) marks an outlier.

Essential oil yield averaged 3.51% *v*/*w* in 2013, 3.14% *v*/*w* in 2014 and 4.05% *v*/*w* in 2015. The differences among years enlightened by the ANOVA (Table 5) are evidenced by the higher value obtained in the last year. EO yield did not appear to be influenced by the location, nor by the Y x L interaction. A marked influence is instead evident across survey dates within a location. The nature of this effect is explained in the graphs in Figure 4. 

**Figure 5:**
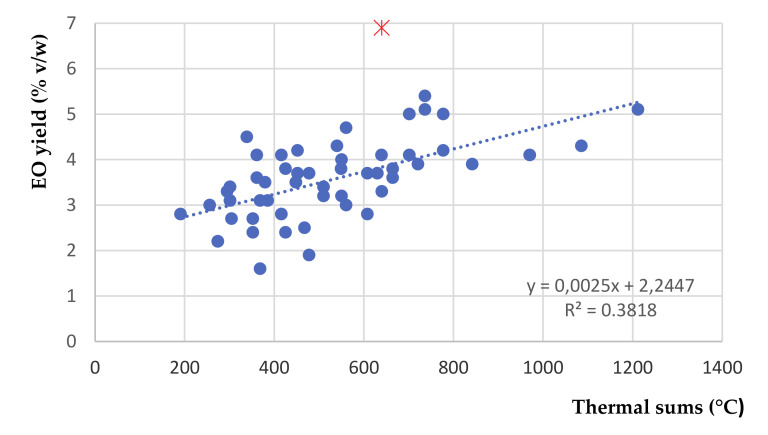
Scatterplot diagram of essential oil yields vs. thermal sums in a 3-year trial on *Origanum heracleoticum* in 3 Sicilian localities. *n* = 53. A red asterisk (*) marks an outlier.

As evidenced, in all years and locations the EO yield shows an increasing trend through all survey dates. Such an effect was striking in farms 1 and 3, whereas it was generally less marked in farm 2, except in 2015 (R^2^ = 0.804). In farm 3, in 2015, an exceptionally high value of EO yield (6.9%) was detected. EO yields evidenced, finally, a fairly good correlation (*r* = 0.618 ***) with thermal sums (Figure 5), and a noticeable (*p*= 0.087) negative correlation (*r* = −0.238) with total rainfall amounts until harvest time.

**Figure 6:**
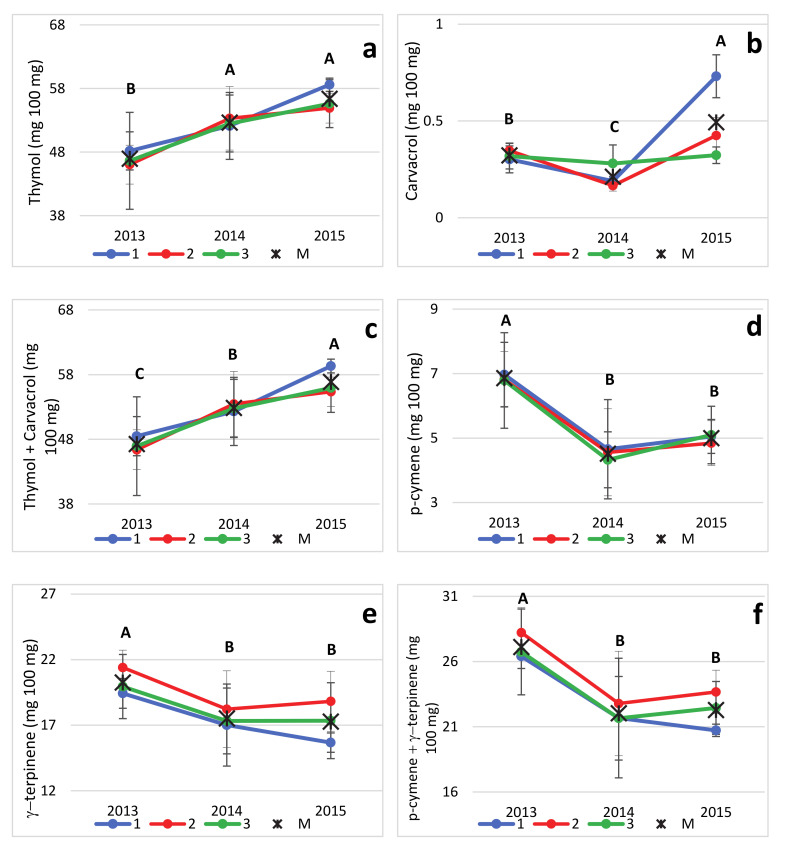

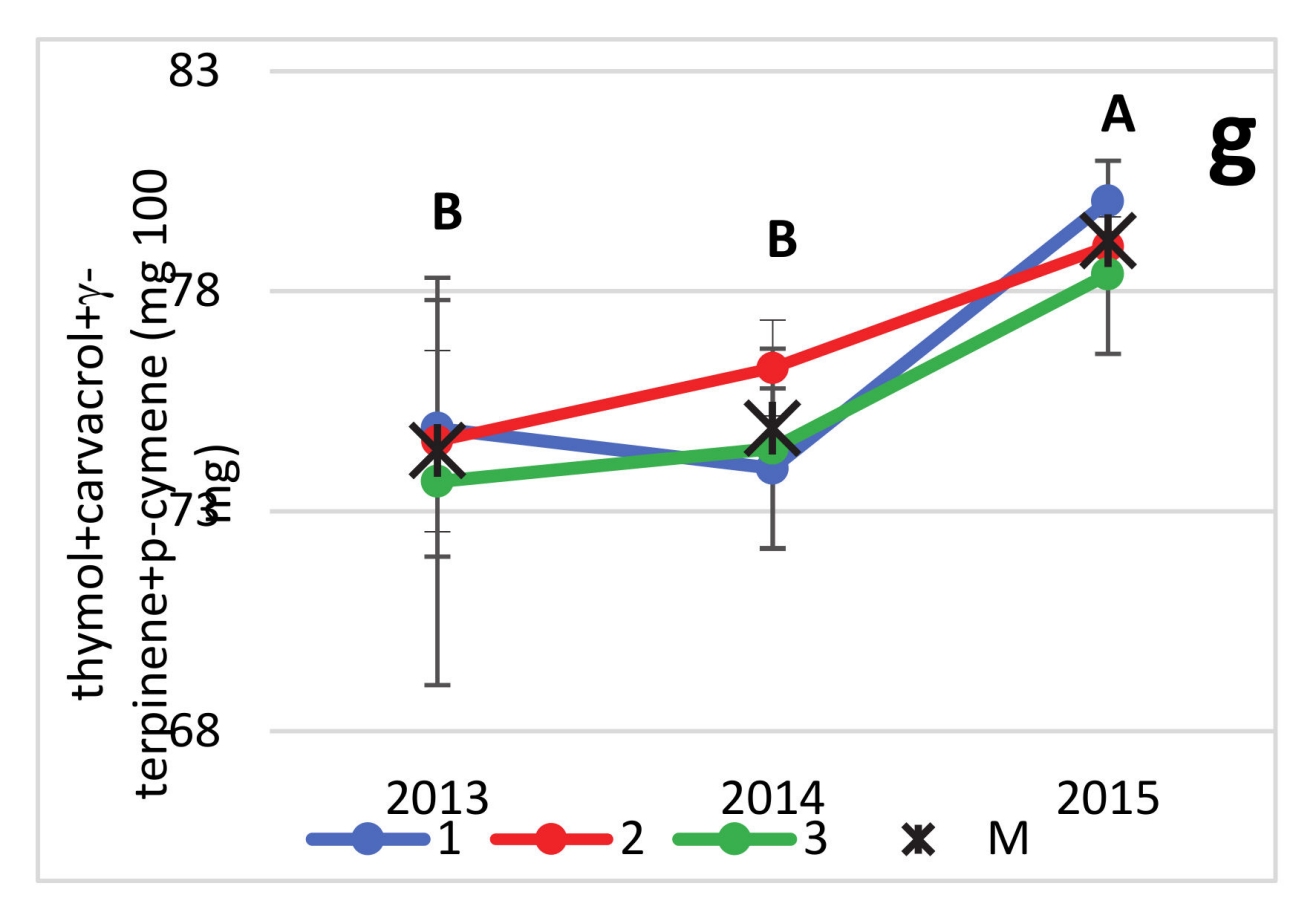
Trends over the years (2013 to 2015) of percentages of thymol (**a**), carvacrol (**b**), thymol + carvacrol (**c**), *p*-cymene (**d**), γ-terpinene (**e**), *p*-cymene + γ-terpinene (**f**) and thymol + carvacrol + *p*-cymene + γ-terpinene (**g**) in the EOs of *Origanum heracleoticum* grown in 3 Sicilian locations (1, 2 and 3). Within each graph, an asterisk (*) indicates the mean year value, and the letters above, when different, indicate significant differences among years at *p* ≤ 0.05 (Tukey’s test).

The variation over years of the major EO components in each locality may be observed in the graphs in Figure 6a–g. As shown, thymol content (Figure 6a) experienced a 20% increase from 2013 to 2015 in all localities. Carvacrol (Figure 6b) followed a less definite pattern, and (as suggested by the high significance of the “Y × L” interaction), the effect over years was different in each location; hence, carvacrol content showed an increase in farm 1 and, to a lesser extent, in farm 2, but remained rather stable in farm 3. Due to the low absolute values reached by carvacrol amount (from 0.16 to 0.73%), the trend of the cumulated values thymol+carvacrol (Figure 6c) was almost identical to that of thymol alone. Oppositely, *p*-cymene and γ-terpinene were characterized by a definite decrease (about 25–29%) throughout the years.

## 3. Discussion

The *Origanum* plants under study proved to belong to the high-yielding, thymol-type biotypes. The values of EO yields and the amounts of the major EO components were similar to those obtained for other oregano cultivations from the same area [10,43,44], and from other areas of Southern [45] and Central Italy [46].

A positive association between thymol and carvacrol was detected. Contrastingly to our results, an inverse correlation between these two compounds was observed by several authors [45,47]. This discrepancy could probably be explained by the fact that, in those experiments, different genotypes were analyzed: since both thymole-type (dealing with low carvacrol) and carvacrol-type (with a low thymol presence) *Origanum* plants were taken into account, a pooled regression including both biotypes shall reasonably return the result of an inverse association between the two compounds. Our results suggest, instead, that in the same genotype the biosynthesis of either compounds is not mutually exclusive, but rather related to the outcomes of alternative pathways; the existence of two independent hydroxylases for thymol and carvacrol, respectively, as previously suggested by Novak et al. [30], seems a plausible hypothesis. The finding that, in a carvacrol-type *O. vulgare* subsp. *hirtum*, thymol and carvacrol are produced by different glands on leaf surface [48], would probably support the existence of separate biosynthetic pathways for the two phenols.

The biosynthetic linkage between thymol and carvacrol, on one side, and *p*-cymene and γ-terpinene, on the other side, seems to be confirmed by the high inverse correlation between their respective total amounts. The ratio between these two chemical groups complies with the current hypothesis that indicates thymol (and carvacrol) as the final product of the biosynthetical route γ-terpinene–*p*-cymene–thymol with the involvement of cytochrome P450 monooxygenases [12,29,49].

In each location, EO yields were found to increase throughout survey dates. An increase in EO yield throughout plant ontogenesis was observed by other researchers as well. As an example, increasing EO levels throughout the flowering stages of *Origanum* vulgare plants were found by Król et al. [40] on a carvacrol-type and by Putievsky et al. [50] on a thymol-type. However, since in the latter trial many non-flowering plants were standing in the experimental plots, and in these non-flowering individuals the EO was increasing as well, the authors hypothesized that such an increase was not to attribute to each plant’s development stage, but rather to the higher temperatures and the photoperiod. In our experiment, the association between thermal sums and oil yields suggests that this positive effect is probably associated with the increasing temperatures along survey dates. No significant effect of photoperiod could be advocated in our case, since all six survey dates occurred in proximity to the summer solstice (21 June), with a very narrow variation among measured daylight durations (from 14:22:00 hh daylight on 21 May, to 14:44:00 hh on 29 June).

This section may be divided by subheadings. It should provide a concise and precise description

A noticeable negative correlation was found between EO yields and rainfall amounts until harvest time. A similar inverse association was found in *O. vulgare* subsp. *hirtum* by Ninou et al. [51], who found the highest EO content in plants cultivated at 40% and 60% of total soil water holding capacity, suggesting a role of EO in water stress adaptability.

Significant variations were found among many EO components, both across years and throughout harvest dates within locations. Variations among years were found in almost all detected compounds: for example, thymol mean content increased from 46.9% (2013) to 56.4% (2015), *p*-cymene mean content decreased from 6.9% (2013) to 5.0% (2015) and so on. This was not surprising, since in most Mediterranean environments, where a high level of diversity is present, phytochemical interannual variability may be considered one of the expressions of plants’ phenotypic plasticity, an adaptive strategy adopted by plants to tolerate the short-term variability of their growth environment [52]. Actually, none of the interannual variations was as high as to allow the shifting from one chemotype to the other: that means, phenotypic variations of chemical profile are always constrained by the genotype.

## 4. Materials and Methods

### 4.1. Plant Material

**Figure 7:**
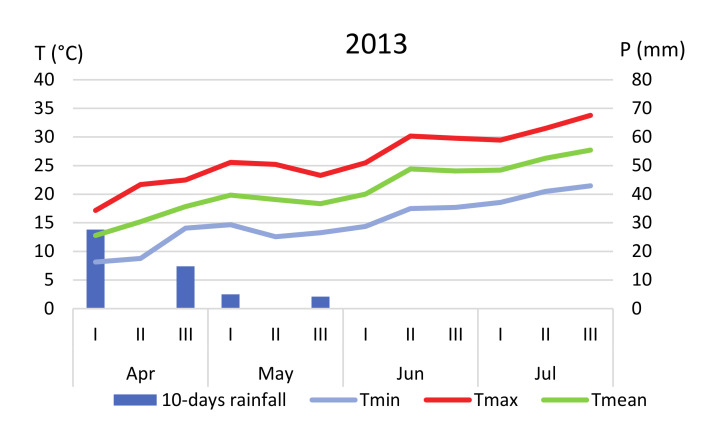

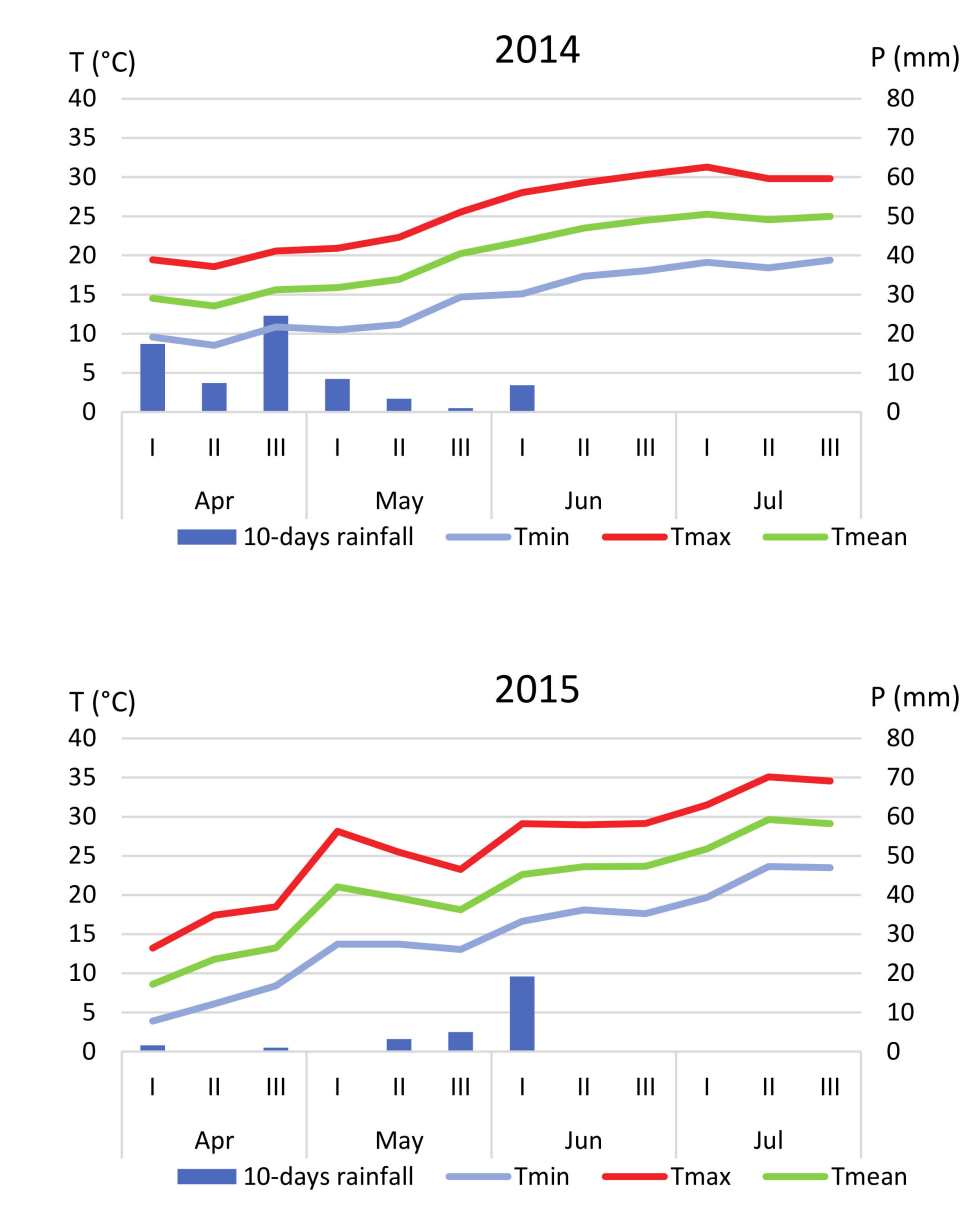
Ten-day values of rainfall (mm) and temperatures (°C) recorded from April to July in 2013, 2014 and 2015 in the meteorological station “Scibica” (farms 1 and 3).

**Figure 8:**
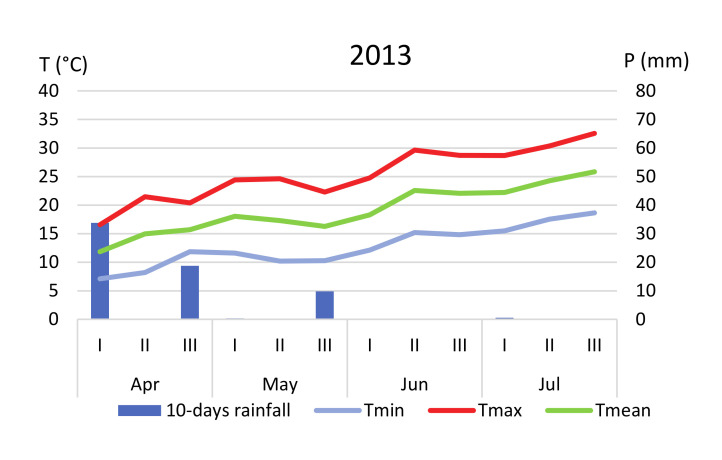

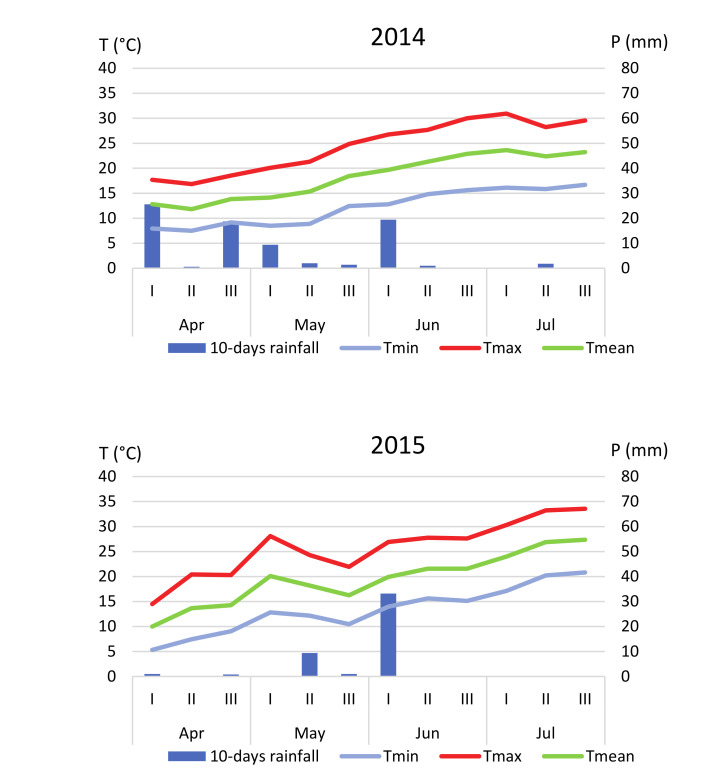
Ten-days values of rainfall (mm) and temperatures (°C) recorded from April to July in 2013, 2014 and 2015 in the meteorological station “Canicattì” (farm 2).

Plant material used for this study was obtained from three farms located in Southern Sicily (Figure 1). In this area, soils are typically clayey (>50% clay) and the predominant climatic pattern is classed as warm Mediterranean [53]. Rainfall is usually distributed between winter and early spring; there are high summer temperatures (maximum values always >30°C) and mild temperatures in winter (rarely falling below 0 °C). Daily values of rainfall and temperatures (Figure 7 and Figure 8) throughout the trials were obtained from the Agrometeorology Service Network of the Sicilian Region [54], by selecting for each farm the nearest meteorological station (“Scibica,” 13°32′54.7″, 37°20′20.9″ N for farms 1 and 3, and “Canicattì,” 13°46′21.6″ E, 37°21′24.3″ N for farm 2).

In all trial years, daily measurements of air temperature were used to calculate the thermal sums (TS) [55] for each farm and harvest date, according to the formula:

(1) $$\mathrm{TS}=\sum_{i=1}^{k}{\left(T_{\mathrm{avg}}\;–T_{\mathrm{base}}\right)}_{i}$$

where:

*i* and *k*: starting and ending date of each calculation; the first day of measurement was set on the 1st of April of each year, whereas the last day was that when each harvest was made.
T_avg_: daily average temperature; and T_base_: base temperature—i.e., temperature value below which plant growth is assumed to be zero. In lack of specific indications for oregano, T_base_ was set at 10 °C.

In all three farms, oregano was cultivated according to the cropping management traditionally applied to this species in Sicily: propagation material was obtained by stock division method from a local wild genotype previously classified as *Origanum vulgare* subsp. *viridulum* (Martrin-Donos) Nyman) (= *O. heracleoticum* L.). Previously prepared rootings were transplanted in the field in winter 2010, allowing distances of 1.0 × 0.5 m between individuals, thereby achieving a plant population of 20,000 plants ha^−1^. Fields were organically managed: one organic fertilization was performed prior to transplant, by distributing 2 t ha^−1^ of cattle manure, further buried by means of a medium-depth (about 40 cm) soil work. No chemical pesticide was used, and weeding was performed by means of one or two shallow soil works (about 5 cm deep) in the early spring of each year. In all three farms, 6 weekly samplings were made between the end of May and the first half of June of each year, i.e., from the start of flowering to the full flowering stage, that being the last moment that when oregano is usually collected in Sicily. Samplings that were started as plants were 3 years old (late spring 2013), and repeated in the following two years (2014 and 2015). Sampled plants (about 0.5 kg per each survey date) were cut up to 5 cm above soil level, and then air-dried in the shadow at an air temperature between 25 and 30 °C, for about 10–15 days. The dried plant material was manually sorted into stems, leaves and flowers. Stems were excluded from subsequent analyses due to their acknowledged low EO content [56]; all extractions and analyses therefore refer to the dried flowering tops (including flowers and leaves) of the plants.

### 4.2. Isolation of the Essential Oil

Air dried 100 g samples of leaves and flowers were subjected to hydrodistillation in a Clevenger-type apparatus until no significant volume increase was recorded in the collected oil (about 3 h). Each essential oil (EO) was dried over anhydrous sodium sulphate and stored under N_2_ in a sealed vial until analysis.

### 4.3. GC and GC–MS Analyses of Essential Oils

Gas chromatographic (GC) analyses were run on a Shimadzu gas chromatograph, Model 17-A equipped with a flame ionization detector (FID), and with operating software Class VP Chromatography Date System version 4.3 (Shimadzu). Analytical conditions: SPB-5 capillary column (15 m × 0.10 mm × 0.15 µm), helium as carrier gas (1 mL/min). Injection in split mode (1:200), injected volume 1 µL (4% essential oil/CH_2_Cl_2_ *v*/*v*), injector and detector temperature 250 and 280 °C, respectively. Linear velocity in column 19 cm/sec. The oven temperature was held at 60 °C for 1 min, and then programmed from 60 to 280 °C at 10 °C/min; then 280 °C for 1 min. Each oil sample was analyzed in triplicate. Percentages of compounds were determined from their peak areas in the GC-FID profiles.

Gas–chromatography-mass spectrometry (GC–MS) was carried out in the fast mode on a Shimadzu GC–MS mod. GCMS-QP5050A, with the same column and the same operative conditions used for analytical GC–FID, operating software GCMS solution version 1.02 (Shimadzu). Ionization voltage 70 eV, electron multiplier 900 V, ion source temperature 180 °C. Mass spectra data were acquired in the scan mode in *m*/*z* range 40–400. The same oil solutions (1 µL) were injected with the split mode (1:96).

### 4.4. Identification of Components of Essential Oils

The identities of components were based on their GC retention indeces (relative to C_9_-C_20_ *n*-alkanes on the SPB-5 column), computer matching of spectral MS data with those from NIST MS libraries [57] and the comparison of the fragmentation patterns with those reported in literature [42]. Internal standards were not used.

### 4.5. Statistical Analysis

In order to detect any relationship between all obtained analytical data, the EO yields (in % *v*/*w*) and the main climatic parameters, a preliminary correlation analysis was carried out. The statistical package PAST 4.03 [58] was used with this purpose.

A more detailed analysis of each compound, taking into consideration the inherent variations over time, was performed by means of variance analysis (ANOVA) using the statistical software Minitab^®^ version 17.1.0 (Minitab Inc., State College, PA, USA, 2013). A preliminary Ryan–Joiner’s test for normality was run for each dataset, and whenever normality hypothesis was rejected, data were submitted to Box-Cox transformation according to the formula Y’ = 1/√(Y). All the above procedures were implemented in the Minitab package. On transformed data, the GLM (General Linear Model) procedure was used, setting as dependent variables the measured analytical data obtained in all experiments, whereas the independent variables were “year,” “location” and “survey date,” respectively. To analyze the trend in time of the chemical profile of each field, a repeated measurement analysis was carried out [59,60], “year” and “location” being set as fixed factors, whereas the “survey date” was considered random, and nested within the “location” factor. When the ANOVA offered statistically significant results, the differences between mean values were appreciated through the Tukey’s test (*p* ≤ 0.05) [61].

## 5. Conclusions

In Sicily, oregano can be considered as a convenient alternative to the traditional crops, as it allows a diversification of products, and an opportunity for new occupational perspectives and income integration [62]. Although this species is mostly used for seasoning of foods, its major appeal comes nowadays from the possibility to use it as a raw matter for producing food flavorings or additives dealing with a higher degree of transformation (and therefore a higher market price) with respect to fresh herbs, developing in this way small, local agro-food industries.

As in all medicinal and aromatic plants (MAPs), in oregano the achievement of a high-quality product is unanimously reputed an indispensable goal of cultivation [62,63,64]. However, great variability exists around the quality parameters that are required by the different industrial and market sectors, and the prevalence (or absence) of specific EO components may be crucial in assessing the commercial success of the crop. For example, thymol is considered the major compound responsible for the antioxidant and antimicrobial actions of oregano, and therefore, when the crop is made to suit industrial purposes, a high yield of this compound is highly desirable. In this case, all technical choices leading to an increase of thymol content will be highly desirable as well. It appears that the choice of the most proper cropping technique, including the scheduling and management of harvest, is a crucial decision for farmers. In the tested Sicilian biotype, a delayed harvest time will be the best option whenever the highest amount of high-thymol EO is required.

Of course, many other aspects of oregano cropping technique are still yet to be verified, in compliance with the various “quality” targets that the different market and industrial may set.

## Figures and Tables

**Table 1:** *Origanum heracleoticum*. 2013. Essential oil (EO) yield and composition (relative peak area percent) in plants cultivated in 3 Sicilian farms (1 to 3) in 6 harvest times (I to VI).

				Farm 1						Farm 2						Farm 3					
				I	II	III	IV	V	VI	I	II	III	IV	V	VI	I	II	III	IV	V	VI
			***EO yield (% v/w)***	*3.1*	*2.4*	*1.9*	*3.0*	*3.6*	*5.0*	*3.3*	*4.5*	*3.5*	*3.5*	*4.3*	*3.3*	*1.6*	*3.8*	*3.7*	*4.7*	*3.8*	*4.2*
**# ^a^**	**RI ^b^**	**RI ^c^**	**Class/Compound**																		
			**Monoterpene hydrocarbons**	**38.72**	**35.79**	**38.37**	**37.59**	**37.84**	**40.94**	**40.10**	**39.97**	**36.92**	**39.25**	**41.64**	**46.13**	**40.03**	**36.63**	**39.76**	**35.41**	**31.79**	**47.62**
2	926	930	α-thujene	1.46	1.41	1.09	1.39	1.61	1.77	1.45	1.58	1.00	1.58	1.83	1.87	1.27	1.51	1.41	1.53	1.46	2.23
3	934	939	α-pinene	0.63	0.65	0.51	0.61	0.69	0.75	0.62	0.65	0.49	0.67	0.76	0.77	0.56	0.63	0.59	0.63	0.59	0.87
4	949	954	camphene	0.07	0.07	0.07	0.08	0.08	0.09	0.07	0.07	0.06	0.08	0.09	0.09	0.06	0.07	0.07	0.07	0.07	0.10
5	972	975	sabinene	0.04	0.05	0.02	0.06	0.15	0.16	0.05	0.15		0.08	0.18	0.17	0.02	0.06	0.12	0.08	0.15	0.24
6	976	979	β-pinene	0.14	0.13	0.12	0.14	0.15	0.16	0.14	0.15	0.12	0.15	0.17	0.17	0.12	0.14	0.14	0.14	0.13	0.20
9	987	991	myrcene	2.23	2.17	1.99	2.24	2.43	2.69	2.23	2.35	2.18	2.42	2.75	2.74	2.01	2.23	2.30	2.34	2.09	3.12
10	1000	1003	α-phellandrene	0.30	0.29	0.23	0.29	0.33	0.36	0.26	0.30	0.31	0.32	0.38	0.37	0.23	0.28	0.31	0.30	0.30	0.42
11	1008	1004	*p*-mentha-1(7),8-diene	0.08	0.09	0.07	0.08	0.09	0.10	0.08	0.09	0.08	0.09	0.10	0.10	0.08	0.08	0.09	0.09	0.08	0.10
12	1016	1017	α-terpinene	3.11	2.83	2.18	2.81	3.10	3.35	2.63	3.06	3.22	3.10	3.62	3.60	2.24	2.86	3.18	2.95	2.90	4.13
13	1028	1025	*p*-cymene	6.16	5.61	8.77	7.05	6.82	7.42	7.47	6.15	5.56	7.18	6.42	8.13	9.38	6.47	6.20	6.24	4.62	7.79
14	1032	1030	limonene	0.47	0.46	0.41	0.46	0.50	0.55	0.47	0.49	0.49	0.50	0.56	0.57	0.42	0.47	0.49	0.49	0.44	0.65
15	1043	1037	*cis*-ocimene	2.29	2.06	4.41	3.01	2.74	2.79	2.97	2.52	2.10	2.71	3.05	3.04	4.17	1.90	2.37	2.09	1.69	3.06
16	1055	1050	*trans*-ocimene	0.38	0.32	0.67	0.45	0.41	0.37	0.40	0.43	0.67	0.59	0.58	0.47	0.50	0.29	0.34	0.31	0.27	0.44
17	1072	1060	γ-terpinene	21.27	19.60	17.78	18.85	18.67	20.31	21.22	21.90	20.53	19.72	21.07	23.95	18.90	19.56	22.09	18.07	16.94	24.14
19	1100	1088	terpinolene	0.09	0.08	0.07	0.07	0.08	0.08	0.07	0.08	0.12	0.08	0.09	0.09	0.07	0.08	0.08	0.07	0.07	0.10
			**Oxygenated monoterpenes**	**56.38**	**55.06**	**51.54**	**56.50**	**56.38**	**53.28**	**53.81**	**54.98**	**54.86**	**54.38**	**50.94**	**47.80**	**48.39**	**57.54**	**48.63**	**58.61**	**62.61**	**45.27**
18	1080	1070	*cis*-sabinene hydrate	0.15	0.20	0.29	0.40	0.51	0.61	0.21	0.25		0.44	0.56	0.54	0.21	0.18	0.34	0.45	0.41	0.54
20	1112	1097	linalool	0.45	0.24	0.90	0.48	0.37	0.18	0.57	0.46	0.41	0.52	0.46	0.48	1.40	0.50	0.61	0.37	0.32	0.72
21	1173	1169	borneol	0.09	0.05	0.07	0.09	0.10	0.11	0.06	0.07	0.10	0.10	0.10	0.09	0.05	0.06	0.07	0.08	0.08	0.10
22	1181	1177	terpinen-4-ol	0.59	0.42	0.45	0.44	0.47	0.47	0.53	0.54	0.69	0.47	0.55	0.57	0.47	0.56	0.51	0.42	0.38	0.62
23	1196	1189	α-terpineol	0.05	0.04	0.05	0.05	0.06	0.06	0.04	0.05	0.06	0.06	0.07	0.07	0.08	0.05	0.05	0.06	0.06	0.04
24	1237	1235	thymol methyl ether	1.68	1.09	4.89	1.79	1.55	0.87	2.36	1.41	1.40	1.90	1.70	1.59	4.05	1.36	1.92	1.03	1.10	1.95
25	1247	1245	carvacrol methyl ether	2.58	2.51	2.62	2.93	3.41	3.70	2.48	2.67	3.15	3.09	3.80	3.58	2.46	2.68	2.98	2.88	2.72	3.97
26	1305	1290	thymol	50.33	50.08	41.95	50.03	49.69	47.04	47.13	49.03	48.68	47.39	43.39	40.56	39.28	51.67	41.77	52.87	57.24	36.91
27	1310	1299	carvacrol	0.41	0.39	0.27	0.28	0.24	0.23	0.36	0.41	0.34	0.37	0.32	0.28	0.24	0.32	0.30	0.42	0.24	0.38
28	1356	1352	thymol acetate	0.05	0.04	0.03				0.06	0.08	0.03	0.04		0.05	0.16	0.16	0.10	0.05	0.06	0.04
			**Sesquiterpenes**	**4.05**	**7.89**	**8.37**	**4.95**	**5.10**	**5.10**	**4.28**	**4.15**	**6.61**	**5.10**	**6.37**	**5.39**	**8.16**	**4.84**	**10.14**	**4.89**	**4.90**	**5.98**
29	1379	1377	α-copaene		0.07	0.07	0.02	0.02	0.02			0.04	0.04	0.02	0.02	0.04		0.05	0.02	0.02	0.02
30	1387	1388	β-bourbonene	0.06	0.06	0.23	0.08	0.06	0.04	0.05	0.04	0.06	0.06	0.05	0.06	0.12	0.04	0.09	0.03	0.03	0.06
31	1423	1419	β-caryophyllene	1.05	1.98	2.17	1.31	1.23	1.30	1.00	1.02	1.94	1.30	1.47	1.33	1.54	1.08	2.20	1.14	1.04	1.33
32	1433	1432	β-copaene	0.03	0.05	0.10	0.04	0.03	0.04	0.03		0.03	0.05	0.03	0.04	0.08	0.02	0.06	0.03	0.03	0.04
33	1437	1435	α-*trans*-bergamotene	0.02	0.04	0.03	0.02	0.02	0.03			0.03	0.02	0.03	0.03	0.03		0.05	0.02	0.03	0.03
34	1459	1455	α-humulene	0.13	0.24	0.24	0.16	0.14	0.15	0.12	0.12	0.23	0.15	0.16	0.16	0.18	0.13	0.25	0.14	0.13	0.16
35	1466	1460	*allo*-aromadendrene	0.06	0.09	0.13	0.06	0.05	0.05	0.06	0.05	0.10	0.08	0.08	0.06	0.12	0.06	0.13	0.07	0.05	0.06
36	1480	1480	γ-muurolene	0.10	0.20	0.27	0.11	0.05	0.07		0.84	0.70		0.07	0.07	0.38	0.09	0.15	0.10	0.04	0.05
37	1486	1485	germacrene D	0.64	1.38	1.29	0.93	1.46	1.44	0.34			0.99	1.96	1.42	0.30	0.68	3.12	0.85	1.53	1.79
38	1500	1496	γ-amorphene													0.26	0.04	0.10	0.06	0.05	0.06
39	1500	1499	α-muurolene	0.06	0.10	0.16	0.07	0.19	0.07	0.08		0.10	0.08		0.06	0.11					
40	1512	1506	β-bisabolene	0.90	1.93	1.86	1.07	1.06	1.15	1.13	0.94	1.86	1.16	1.34	1.26	1.76	1.20	2.15	1.19	1.15	1.35
41	1519	1514	γ-cadinene	0.07	0.14	0.17	0.09	0.06	0.08	0.09	0.05	0.19	0.10	0.09	0.08	0.23	0.08	0.13	0.09	0.06	0.06
42	1528	1523	δ-cadinene	0.27	0.48	0.57	0.28	0.22	0.24	0.31	0.22	0.58	0.35	0.34	0.24	0.82	0.32	0.56	0.32	0.21	0.24
43	1538	1535	α-cadinene			0.02				0.03	0.02	0.03				0.04					
44	1565	1563	(*E*)-nerolidol																		
45	1585	1601	spathulenol	0.11	0.14	0.29	0.11	0.06	0.03		0.10		0.10	0.04	0.07	0.27	0.09	0.08	0.07	0.02	0.06
46	1590	1640	globulol	0.13	0.20	0.27	0.18	0.11	0.09	0.26	0.16	0.06	0.13	0.08	0.12	0.36	0.17	0.12	0.13	0.06	0.10
47	1650	1641	*epi*-*a*-muurolol	0.12	0.20	0.09	0.11	0.09	0.07	0.15	0.13	0.17		0.16	0.09	0.30	0.17	0.20	0.14	0.09	0.12
48	1654	1642	cubenol							0.07	0.05	0.03		0.03	0.03	0.08	0.06	0.05	0.05	0.02	0.03
49	1663	1653	α-cadinol	0.22	0.39	0.29	0.21	0.17	0.15	0.27	0.27	0.27	0.27	0.29	0.15	0.52	0.32	0.39	0.26	0.16	0.21
50	1768	1761	*cis*-lanceol	0.09	0.18	0.09	0.10	0.08	0.08	0.15	0.08	0.11	0.11	0.13	0.11	0.61	0.24	0.29	0.16	0.19	0.21
			**Others**	**0.20**	**0.10**	**0.32**	**0.14**	**0.12**	**0.08**	**0.22**	**0.19**	**0.09**	**0.13**	**0.05**	**0.08**	**0.58**	**0.14**	**0.15**	**0.08**	**0.04**	**0.13**
1	852	855	2-(*E*)-hexenal	0.18	0.10	0.22	0.12	0.10	0.06	0.22	0.19	0.09	0.13	0.05	0.08	0.43	0.14	0.13	0.08	0.04	0.13
7	978	978	octen-3-ol			0.04	0.03	0.02	0.02							0.07		0.02			
8	984	984	3-octanone	0.02		0.06										0.05					
			**Monoterpene hydrocarbons**	**38.72**	**35.79**	**38.37**	**37.59**	**37.84**	**40.94**	**40.10**	**39.97**	**36.92**	**39.25**	**41.64**	**46.13**	**40.03**	**36.63**	**39.76**	**35.41**	**31.79**	**47.62**
			**Oxygenated monoterpenes**	**56.38**	**55.06**	**51.54**	**56.50**	**56.38**	**53.28**	**53.81**	**54.98**	**54.86**	**54.38**	**50.94**	**47.80**	**48.39**	**57.54**	**48.63**	**58.61**	**62.61**	**45.27**
			**Sesquiterpenes**	**4.05**	**7.89**	**8.37**	**4.95**	**5.10**	**5.10**	**4.28**	**4.15**	**6.61**	**5.10**	**6.37**	**5.39**	**8.16**	**4.84**	**10.14**	**4.89**	**4.90**	**5.98**
			**Others**	**0.20**	**0.10**	**0.32**	**0.14**	**0.12**	**0.08**	**0.22**	**0.19**	**0.09**	**0.13**	**0.05**	**0.08**	**0.58**	**0.14**	**0.15**	**0.08**	**0.04**	**0.13**

^a^ The numbering refers to elution order, and values (relative peak area percent) represent averages of 3 determinations; ^b^ retention index (RI) relative to standard mixture of *n*-alkanes on SPB-5 column. ^c^ Literature retention index (RI) [42].

**Table 2:** *Origanum heracleoticum*. 2014. EO yield and composition (relative peak area percent) in plants cultivated in 3 Sicilian farms (1 to 3) in 6 harvest times (I to VI).

				Farm 1						Farm 2						Farm 3					
				I	II	III	IV	V	VI	I	II	III	IV	V	VI	I	II	III	IV	V	VI
			***EO yield (% v/w)***	*2.2*	*2.4*	*2.8*	*3.2*	*2.8*	*4.1*	*2.8*	*3.0*	*2.7*	*3.1*	*2.5*	*3.8*	*2.2*	*2.7*	*4.1*	*3.4*	*3.7*	*5.0*
**# ^a^**	**RI ^b^**	**RI ^c^**	**Class/Compound**																		
			**Monoterpene hydrocarbons**	**19.70**	**27.38**	**29.63**	**28.61**	**36.88**	**40.23**	**23.69**	**24.96**	**36.25**	**38.68**	**29.57**	**36.42**	**24.90**	**27.85**	**37.24**	**31.95**	**27.54**	**37.94**
2	926	930	α-thujene	0.62	0.88	0.84	1.16	1.14	1.50	0.74	0.76	1.09	1.29	0.82	1.22	0.74	0.95	1.40	1.27	1.23	1.43
3	934	939	α-pinene	0.27	0.40	0.37	0.49	0.50	0.63	0.32	0.33	0.46	0.55	0.37	0.52	0.32	0.40	0.56	0.51	0.49	0.59
4	949	954	camphene	0.03	0.05	0.05	0.06	0.06	0.08	0.04	0.04	0.06	0.07	0.05	0.06	0.04	0.05	0.07	0.06	0.06	0.07
5	972	975	sabinene	t	t	t	t	t	t	0.10	0.10	0.14	0.15	0.10	t	0.08	0.12	0.18	0.16	0.14	0.15
6	976	979	β-pinene	0.08	0.11	0.11	0.13	0.14	0.16	0.07	0.08	0.11	0.12	0.09	0.13	0.07	0.09	0.13	0.12	0.11	0.14
9	987	991	myrcene	1.01	1.39	1.50	1.72	2.03	2.52	1.21	1.26	1.91	2.13	1.44	2.12	1.20	1.50	2.39	2.01	1.80	2.38
10	1000	1003	α-phellandrene	0.15	0.20	0.22	0.24	0.28	0.36	0.18	0.19	0.28	0.32	0.21	0.29	0.17	0.21	0.34	0.29	0.26	0.34
11	1008	1004	*p*-mentha-1(7),8-diene	0.04	0.06	0.06	0.06	0.08	0.09	0.05	0.05	0.07	0.08	0.06	0.07	0.05	0.06	0.09	0.08	0.06	0.09
12	1016	1017	α-terpinene	1.50	2.07	2.25	2.43	2.82	3.46	1.92	1.95	2.90	3.15	2.10	2.86	1.89	2.15	3.41	2.83	2.47	3.37
13	1028	1025	*p*-cymene	2.59	3.72	4.12	4.09	6.89	6.52	2.68	3.00	4.70	5.56	5.44	6.14	2.75	3.83	4.98	4.52	4.40	5.48
14	1032	1030	limonene	0.23	0.31	0.33	0.37	0.42	0.55	0.27	0.29	0.42	0.46	0.33	0.43	0.26	0.33	0.50	0.43	0.39	0.51
15	1043	1037	*cis*-ocimene	1.37	1.85	2.03	1.83	2.36	2.58	1.29	1.56	2.28	2.17	1.70	2.46	1.71	2.08	1.92	1.87	1.63	2.15
16	1055	1050	*trans*-ocimene	0.17	0.25	0.28	0.26	0.33	0.38	0.17	0.31	0.41	0.54	0.34	0.39	0.23	0.27	0.28	0.27	0.24	0.39
17	1072	1060	γ-terpinene	11.59	16.04	17.42	15.71	19.75	21.33	14.60	14.98	21.35	22.02	16.47	19.66	15.35	15.74	20.91	17.49	14.18	20.77
19	1100	1088	terpinolene	0.06	0.06	0.07	0.07	0.08	0.09	0.05	0.06	0.08	0.08	0.07	0.08	0.05	0.06	0.09	0.07	0.08	0.09
			**Oxygenated monoterpenes**	**64.58**	**61.14**	**59.70**	**62.62**	**53.97**	**49.92**	**65.73**	**65.58**	**54.97**	**53.48**	**59.84**	**55.81**	**63.03**	**59.39**	**51.82**	**59.37**	**64.05**	**54.98**
18	1080	1070	*cis*-sabinene hydrate	0.28	0.36	0.32	0.31	0.33	0.45	0.36	0.28	0.25	0.31	0.45	0.35	0.30	0.29	0.38	0.38	0.34	0.34
20	1112	1097	linalool	0.44	0.46	0.48	0.35	0.35	0.31	0.29	0.40	0.36	0.32	0.41	0.33	0.42	0.56	0.34	0.33	0.41	0.24
21	1173	1169	borneol	0.08	0.11	0.03	0.09	0.09	0.14	0.08		0.08	0.09	0.03	0.02	0.09	0.07	0.08	0.08	0.08	0.09
22	1181	1177	terpinen-4-ol	0.25	0.48	0.19	0.49	0.22	0.60	0.36	0.45	0.45	0.44	0.25	0.21	0.40	0.24	0.56	0.41	0.22	0.52
23	1196	1189	α-terpineol	0.06	0.03	0.04	0.06	0.03	0.07	0.05	0.06	0.06	0.06	0.03	0.03	0.06	0.06		0.06	0.03	0.07
24	1237	1235	thymol methyl ether	2.59	2.55	2.01	1.66	2.18	1.25	2.08	2.13	2.17	2.07	2.28	1.09	2.26	2.94	1.53	1.50	1.43	0.85
25	1247	1245	carvacrol methyl ether	2.39	2.70	2.83	2.80	3.04	3.85	2.32	2.44	2.63	2.78	3.19	3.02	2.01	2.65	3.49	2.83	3.03	3.27
26	1305	1290	thymol	58.09	54.08	53.52	56.58	47.44	43.00	59.87	59.48	48.67	47.19	52.85	50.43	57.13	52.27	44.88	53.48	57.91	49.28
27	1310	1299	carvacrol	0.19	0.22	0.18	0.20	0.15	0.20	0.17	0.21	0.19	0.13	0.15	0.15	0.20	0.21	0.34	0.23	0.47	0.23
28	1356	1352	thymol acetate	0.19	0.06	0.07	0.03	0.03	0.05	0.16	0.06	0.08	0.04	0.06	0.04	0.12	0.09	0.10	0.06	0.03	0.04
			**Sesquiterpenes**	**13.29**	**10.49**	**9.26**	**8.14**	**8.01**	**9.06**	**9.75**	**8.86**	**7.96**	**7.52**	**9.38**	**6.97**	**11.09**	**11.43**	**9.88**	**7.91**	**8.08**	**6.79**
35	1379	1377	α-copaene	0.04	0.04	0.04	0.03	0.04		t		0.03	0.03			0.04	0.03	0.03	0.03	0.03	0.03
36	1387	1388	β-bourbonene	0.08	0.10	0.09	0.07			0.05	0.08	0.07	0.06		0.06	0.10	0.10	0.05	0.06	0.06	0.03
38	1425	1419	β-caryophyllene	2.41	2.28	2.04	1.76	1.90	2.08	1.87	1.89	1.77	1.79	2.22	1.64	2.30	2.18	1.85	1.60	1.56	1.53
39	1433	1432	β-copaene	0.06	0.05	0.05	0.05	0.05	0.05	0.04	0.04	0.04	0.04	0.06	0.05	0.05	0.05	0.04	0.04	0.04	0.04
40	1437	1435	α-*trans*-bergamotene	0.06	0.05	0.05	0.04	0.04	0.05	0.05	0.05	0.04	0.04	0.05	0.04	0.05	0.05	0.05	0.04	0.04	0.03
42	1460	1455	α-humulene	0.30	0.24	0.21	0.22	0.24	0.27	0.24	0.24	0.22	0.22	0.29	0.20	0.27	0.26	0.20	0.20	0.20	0.20
43	1467	1460	*allo*-aromadendrene	0.18	0.12	0.09	0.08	0.10	0.11	0.11	0.10	0.08	0.08	0.10	0.08	0.14	0.16	0.11	0.08	0.10	0.08
44	1480	1480	γ-muurolene	0.08	0.07	0.04	0.07	0.07	0.13	0.04	0.06	0.04	0.05	0.09	0.10	0.08	0.06	0.05	0.05	0.06	0.09
45	1488	1485	germacrene D	4.11	3.27	3.17	2.56	2.49	2.76	3.37	2.55	2.48	1.88	2.34	2.08	3.51	3.63	3.22	2.62	2.48	1.86
46	1502	1500	bicyclogermacrene	0.41	0.29	0.21	0.19	0.20	0.07	0.25	0.23	0.18	0.15	0.16	t	0.32	0.42	0.23	0.18	0.18	0.12
47	1512	1506	β-bisabolene	3.05	2.30	2.01	1.78	1.81	2.35	2.26	2.00	1.83	1.85	2.38	1.57	2.30	2.29	2.31	1.74	1.68	1.58
48	1519	1514	γ-cadinene	0.12	0.08	0.07	0.08	0.08	0.14	0.07	0.07	0.06	0.07	0.14	0.14	0.09	0.09	0.11	0.06	0.09	0.14
49	1528	1523	δ-cadinene	0.60	0.37	0.32	0.32	0.32	0.47	0.34	0.32	0.29	0.28	0.39	0.34	0.41	0.46	0.41	0.28	0.35	0.37
50	1538	1535	α-cadinene											0.08	0.06						
51	1543	1535	*trans*-cadina-1(2)4-diene		0.08		0.06	0.06		0.08	0.07	0.06	0.06	0.02	0.09	0.08	0.08	0.08	0.06	0.06	0.06
52	1565	1563	*E*-nerolidol	0.04	0.03	0.03	0.03	0.06						0.05	0.03		0.04	0.02			
53	1580	1590	germacrene D-4ol	0.18	0.15	0.11	0.09	0.12	0.15	0.18	0.12	0.09	0.09	0.14	0.09	0.15	0.18	0.16	0.15	0.11	0.10
54	1585	1601	spathulenol	0.06	0.06	0.04	0.04	0.02	0.07	0.05	0.03	0.03	0.03	0.02	0.03	0.06	0.05	0.02	0.03	0.03	0.05
55	1590	1602	caryophyllene oxide	0.10	0.10	0.09	0.07	0.02			0.07	0.06	0.06	0.03		0.11	0.08	0.05	0.05	0.07	0.09
56	1590	1640	globulol	0.06	0.04	0.03	0.03	0.03				0.02	0.03	0.04		0.04	0.04	0.03	0.02	0.03	0.16
57	1635	1641	*epi*-α-muurolol	0.50	0.15	0.13	0.11	0.20		0.15	0.12			0.21	0.16	0.17	0.19			0.12	
58	1654	1642	cubenol			0.02		0.04	0.13				0.09	0.03			0.03	0.15	0.11		
59	1663	1653	α-cadinol	t	0.29	0.24	0.19	t	0.23	0.29	0.23	0.20	0.16	0.06	0.04	0.35	0.35	0.29	0.21	0.23	0.02
60	1695	1689	eudesma-4(15),7-dien-1-β-ol	0.05	0.04	0.04	0.03									0.04	0.04	0.02	0.02	0.03	0.06
61	1747	1741	mint sulfide	0.04		0.04		0.14			0.32	0.15			0.16		0.04	0.03		0.20	0.12
62	1754	1746	α-sinensal	0.09	0.04	0.04	0.03						0.30	0.24		0.04	0.06	0.04	0.03		
63	1768	1761	*cis*-lanceol	0.66	0.23	0.03	0.16			0.27	0.22	0.20	0.12	0.22		0.35	0.44	0.31	0.22	0.30	
			**Others**	**0.24**	**0.29**	**0.24**	**0.21**	**0.27**	**0.15**	**0.09**	**0.19**	**0.21**	**0.10**	**0.17**	**0.18**	**0.16**	**0.17**	**0.09**	**0.06**	**0.05**	**0.03**
1	855	867	α-hexenal	0.12	0.20	0.16	0.10	0.12	t	0.09	0.19	0.21	0.10	0.13	0.06	0.16	0.07	0.07	0.06	0.05	0.03
7	978	978	octen-3-ol	0.06	0.09	0.09	0.11	0.11	0.15	t	t	t	t	t	0.12	t	t	t	t	t	t
8	984	984	3-octanone	t	t	t	t	t	t	t	t	t	t	t	t	t	0.04	t	t	t	t
10	994	999	3-octanol	t	t	t	t	t	t												
18	1042	1035	phenylacetaldehyde	0.03						t	t	t	t	t	t	t	0.04	0.02	t	t	t
34	1361	1367	eugenol	0.03				0.04						0.04			0.02				
			**Monoterpene hydrocarbons**	**19.70**	**27.38**	**29.63**	**28.61**	**36.88**	**40.23**	**23.69**	**24.96**	**36.25**	**38.68**	**29.57**	**36.42**	**24.90**	**27.85**	**37.24**	**31.95**	**27.54**	**37.94**
			**Oxygenated monoterpenes**	**64.58**	**61.14**	**59.70**	**62.62**	**53.97**	**49.92**	**65.73**	**65.58**	**54.97**	**53.48**	**59.84**	**55.81**	**63.03**	**59.39**	**51.82**	**59.37**	**64.05**	**54.98**
			**Sesquiterpenes**	**13.29**	**10.49**	**9.26**	**8.14**	**8.01**	**9.06**	**9.75**	**8.86**	**7.96**	**7.52**	**9.38**	**6.97**	**11.09**	**11.43**	**9.88**	**7.91**	**8.08**	**6.79**
			**Others**	**0.24**	**0.29**	**0.24**	**0.21**	**0.27**	**0.15**	**0.09**	**0.19**	**0.21**	**0.10**	**0.17**	**0.18**	**0.16**	**0.17**	**0.09**	**0.06**	**0.05**	**0.03**

^a^ The numbering refers to elution order, and values (relative peak area percent) represent averages of 3 determinations; ^b^ retention index (RI) relative to standard mixture of *n*-alkanes on SPB-5 column. ^c^ Literature retention index (RI) [42].

**Table 3:** *Origanum heracleoticum*. 2015. EO yield and composition (relative peak area percent) in plants cultivated in 3 Sicilian farms (1 to 3) in 6 harvest times (I to VI).

				Farm 1						Farm 2						Farm 3					
				I	II	III	IV	V	VI	I	II	III	IV	V	VI	I	II	III	IV	V	VI
			***EO yield (% v/w)***	*3.1*	*3.6*	*3.7*	*3.2*	*4.1*	*5.1*	*3.7*	*3.9*	*3.9*	*4.1*	*4.3*	*5.1*	*3.4*	*4.1*	*4.2*	*4.0*	*6.9*	*5.4*
# ^a^	**RI ^b^**	**RI ^c^**	**Class/Compound**																		
			**Monoterpene hydrocarbons**	**31.54**	**30.14**	**31.41**	**30.72**	**31.54**	**32.56**	**39.03**	**35.64**	**35.04**	**33.83**	**34.51**	**33.61**	**37.08**	**35.38**	**34.04**	**32.70**	**32.07**	**30.92**
2	931	930	α-thujene	1.44	1.51	1.60	1.50	1.66	1.84	1.56	1.51	1.59	1.57	1.60	1.59	1.51	1.51	1.60	1.65	1.64	1.59
3	939	939	α-pinene	0.58	0.60	0.64	0.60	0.66	0.70	0.63	0.61	0.62	0.65	0.65	0.66	0.61	0.61	0.64	0.65	0.67	0.66
4	954	954	camphene	0.06	0.07	0.07	0.07	0.08	0.08	0.07	0.07	0.07	0.08	0.07	0.07	0.07	0.07	0.07	0.08	0.08	0.07
5	977	975	sabinene	0.12	0.11	0.11	0.10	0.11	0.13	0.13	0.14	0.14	0.10	0.13	0.11	0.13	0.13	0.13	0.15	0.10	0.09
6	981	979	β-pinene	0.12	0.12	0.13	0.13	0.15	0.16	0.13	0.13	0.13	0.14	0.14	0.14	0.14	0.13	0.14	0.15	0.15	0.15
8	993	991	myrcene	2.06	2.13	2.25	2.08	2.25	2.42	2.25	2.22	2.32	2.27	2.31	2.29	2.16	2.21	2.26	2.35	2.31	2.28
9	1007	1003	α-phellandrene	0.29	0.29	0.31	0.27	0.30	0.33	0.32	0.32	0.32	0.31	0.32	0.31	0.30	0.31	0.32	0.32	0.31	0.30
10	1014	1004	pseudolimonene	0.08	0.07	0.08	0.08	0.08	0.08	0.08	0.08	0.08	0.08	0.08	0.08	0.07	0.08	0.08	0.08	0.08	0.08
11	1021	1017	α-terpinene	2.83	2.77	2.87	2.62	2.87	3.10	3.41	3.17	3.16	2.93	3.05	2.88	3.13	3.18	3.15	2.96	2.82	2.66
12	1030	1025	*p*-cymene	4.16	4.56	5.18	5.26	5.55	5.60	3.90	4.16	4.55	5.35	5.28	5.83	4.13	4.09	4.47	5.62	6.04	6.23
13	1034	1030	limonene	0.42	0.42	0.46	0.42	0.46	0.49	0.45	0.45	0.45	0.45	0.46	0.46	0.43	0.44	0.46	0.47	0.47	0.45
15	1042	1037	*cis*-ocimene	1.74	1.58	1.76	2.16	1.83	1.61	2.14	2.02	2.12	1.96	2.07	1.98	2.35	1.93	1.97	2.26	2.25	2.10
17	1053	1050	*trans*-ocimene	0.31	0.29	0.32	0.38	0.31	0.31	0.65	0.56	0.66	0.79	0.82	0.63	0.42	0.33	0.30	0.37	0.38	0.36
18	1068	1060	γ-terpinene	17.23	15.53	15.55	14.96	15.15	15.61	23.20	20.09	18.72	17.05	17.41	16.47	21.51	20.29	18.34	15.48	14.68	13.78
20	1092	1088	terpinolene	0.08	0.08	0.09	0.09	0.09	0.10	0.09	0.08	0.08	0.09	0.08	0.09	0.08	0.08	0.08	0.09	0.09	0.09
23	1131	1132	*allo*-ocimene	0.02	t	t	0.01	0.01	0.01	0.03	0.03	0.03	0.03	0.02	0.02	0.03	t	0.02	0.03	0.02	0.02
			**Oxygenated monoterpenes**	**62.77**	**65.59**	**64.34**	**64.61**	**64.68**	**64.28**	**55.41**	**58.96**	**61.16**	**62.17**	**61.20**	**61.95**	**54.73**	**58.10**	**60.91**	**63.83**	**64.05**	**65.47**
14	1038	1038	1,8-cineole	0.01	0.01	0.01	0.02	0.01	0.02												
19	1075	1070	*cis*-sabinene hydrate	0.13	0.13	0.20	0.18	0.27	0.25	0.11	0.14	0.18	0.15	0.22	0.24	0.09	0.13	0.17	0.16	0.19	0.20
21	1103	1097	linalool	0.48	0.37	0.38	0.56	0.41	0.30	0.40	0.37	0.41	0.39	0.37	0.29	0.69	0.39	0.41	0.45	0.35	0.28
22	1110	1098	*trans*-sabinene hydrate	0.01	0.02	t	0.02	0.02	0.02										0.02	t	0.02
24	1173	1169	borneol	0.02	0.01	0.04	0.05	0.05	0.03	0.03	0.12	0.02	0.02	0.05	0.04	0.02	0.02	0.03	0.03	0.03	0.06
25	1183	1177	terpinen-4-ol	0.54	0.52	0.51	0.53	0.52	0.56	0.56	0.49	0.20	0.25	0.53	0.45	0.27	0.36	0.24	0.37	0.35	0.49
26	1191	1189	α-terpineol	0.04	0.01	0.03	0.07		0.01	0.06	0.06	0.03	0.03	0.07	0.02	0.06	0.06	0.06	0.02	0.05	0.05
27	1207	1199	γ-terpineol	0.07	0.02	0.05	0.07	0.07	0.08	0.03	0.02	0.07	0.07	0.03	0.07	0.02	0.03	0.04	0.08	0.07	0.05
28	1208	1201	*t*-dihydrocarvone							0.06	t	0.02	0.03	t	0.02			0.03	0.03	0.04	0.03
29	1240	1235	thymol methyl ether	1.59	1.15	1.19	1.82	1.05	0.53	1.15	1.05	1.05	1.19	1.07	0.67	1.94	1.20	1.20	1.54	1.15	0.75
30	1250	1245	carvacrol methyl ether	2.33	2.44	2.61	2.55	2.53	2.48	2.10	2.34	2.68	2.81	2.72	2.87	2.33	2.55	2.83	3.02	2.84	3.12
31	1311	1290	thymol	56.71	60.14	58.61	58.07	59.02	59.02	50.22	53.74	56.14	56.86	55.69	56.89	48.84	52.97	55.59	57.76	58.56	60.00
32	1314	1299	carvacrol	0.73	0.70	0.66	0.63	0.70	0.97	0.56	0.52	0.32	0.33	0.44	0.37	0.34	0.29	0.25	0.34	0.36	0.37
33	1360	1352	thymol acetate	0.09	0.06	0.03	0.03	0.02	0.02	0.13	0.10	0.05	0.03		t	0.13	0.10	0.05	0.02	0.02	0.02
34	1364	1373	carvacrol acetate	0.02	0.01	0.02	0.01	0.01	0.02	0.01								t		0.04	0.03
			**Sesquiterpenes**	**4.52**	**3.50**	**3.62**	**3.80**	**3.20**	**2.70**	**5.05**	**5.06**	**3.50**	**3.63**	**3.83**	**3.67**	**5.70**	**5.81**	**4.59**	**3.16**	**3.49**	**3.44**
35	1385	1375	α-ylangene															0.02		0.05	T
36	1384	1377	α-copaene^d^	0.02	0.01	t	0.02	0.02	0.01	0.04				0.02	0.02	0.02	0.02	0.03	t	t	T
37	1393	1388	β-bourbonene	0.05	0.04	0.04	0.06	0.04	0.02	0.02	0.04	0.03	0.02	0.04	0.03	0.06	0.04	0.03	0.05		
38	1399	1391	β-elemene								0.03	0.02	0.04	0.02	t	0.02	0.03	0.02			
39	1430	1419	β-caryophyllene	0.90	0.76	0.78	0.83	0.76	0.65	1.11	1.08	0.77	0.92	0.95	0.95	1.22	1.19	0.98	0.75	0.87	0.88
40	1438	1432	β-copaene	0.03	0.03	0.03	0.04	0.03	0.02	0.03	0.03	0.02	0.03	0.03	0.03	0.04	0.03	0.03	0.02	0.04	0.04
41	1443	1435	*trans*-α-bergamotene	0.02	0.02	0.02	0.02	0.02	0.02	0.02	0.08	0.02	0.02	0.02	0.02	0.02	0.03	0.02	t	0.02	0.02
42	1463	1455	α-humulene	0.11	0.08	0.09	0.10	0.09	0.08	0.13	0.09	0.10	0.10	0.11	0.11	0.15	0.14	0.12	0.09	0.11	0.09
43	1470	1460	*allo*-aromadendrene	0.05	0.04	0.04	0.05	0.04	0.03	0.05	0.05	0.04	0.05	0.05	0.05	0.06	0.06	0.05	0.04	0.05	0.05
44	1485	1480	γ-muurolene	0.06	0.05	0.06	0.08	0.07	0.06	0.05	0.05	0.05	0.08	0.07	0.09	0.06	0.07	0.06	0.04	0.08	0.09
45	1491	1485	germacrene D	1.25	0.95	0.84	0.78	0.68	0.59	1.28	1.33	0.83	0.65	0.67	0.70	1.66	1.67	1.27	0.82	0.77	0.68
46	1497	1496	γ-amorphene	0.01	0.01	0.01	0.01	0.09	0.01	0.01		t		0.03	0.02	t	t	t	t	0.03	0.04
47	1505	1500	bicyclogermacrene	0.23	0.13	0.14	0.16	0.01	0.01	0.15	0.20	0.11	0.10			0.21	0.16	0.12	0.15	0.03	0.04
48	1515	1506	β-bisabolene	1.01	0.81	0.78	0.84	0.70	0.66	1.07	0.98	0.67	0.80	0.84	0.82	1.11	1.15	0.90	0.59	0.73	0.75
49	1523	1514	γ-cadinene	0.07	0.05	0.07	0.07	0.06	0.06	0.06	0.05	0.05	0.07	0.07	0.09	0.06	0.07	0.06	0.05	t	0.10
50	1532	1523	δ-cadinene	0.22	0.20	0.21	0.23	0.19	0.17	0.25	0.24	0.20	0.22	0.23	0.25	0.26	0.29	0.24	0.18	0.24	0.25
51	1542	1531	*trans*-γ-bisabolene	0.04	0.03	0.03	0.03	0.03	0.03	0.04	0.03	0.02	0.03	0.03	t	0.04	t	t	0.02		
52	1543	1535	*trans*-cadina-1(2),4-diene		0.02						0.02		0.01			0.02		t	0.03		0.02
53	1565	1563	*trans*-nerolidol		0.04						t		0.05			0.05		0.03			0.04
54	1585	1601	guaiol	0.04	0.01	0.03	0.03	0.03	0.02	0.03	0.05	0.04	0.06	0.04	0.04	0.02	0.03	0.04	0.04	0.05	0.05
55	1588	1608	β-atlantol	0.05	0.01	0.04	0.08	0.05	0.02	0.02	0.03	0.02	0.02	0.05	0.02	0.02	0.05	0.03	0.06	0.08	0.04
56	1653	1640	*epi*-α-cadinol	0.12	0.17	0.09	0.10	0.08	0.06	0.11	0.12	0.10	t	0.09	0.09	0.18	0.15	0.12	0.09	0.09	0.08
57	1666	1646	α-muurolol	0.18	0.04	0.16	0.15	0.11	0.09	0.20	0.23	0.17	0.15	0.16	0.14	0.21	0.27	0.21	0.15	0.14	0.13
58	1680	1680	khusinol	0.05	0.01	0.03	0.04	0.03	0.02							0.02	0.07	0.02	t	t	0.02
59	1747	1741	mint sulfide	t	t	0.01	0.02	0.09	0.01	0.16	0.17	0.13	0.13	0.22	0.14	0.02	0.02	0.02	t	0.03	0.06
60	1757	1761	lanceol	0.02	t	0.12	0.07	0.01	0.07	0.21	0.18	0.13	0.08	0.11	0.08	0.18	0.28	0.17	t	0.08	t
			**Others**	**0.17**	**0.07**	**0.06**	**0.09**	**0.04**	**0.04**	**0.10**	**0.05**	**0.04**	**0.03**	**0.05**	**0.03**	**0.10**	**0.04**	**0.03**	**0.04**	**0.04**	**0.02**
1	856	855	2*E*-Hexenal	0.16	0.06	0.05	0.08	0.03	0.04	0.09	0.05	0.04	0.03	0.05	0.03	0.08	0.04	0.03	0.04	0.04	0.02
7	987	984	3-octanone							0.01			t			0.02	t	t	t	t	t
16	1050	1042	phenylacetaldehyde	0.01	0.01	0.01	0.01	0.01	0.01							t	t	t			
			**Monoterpene hydrocarbons**	**31.54**	**30.14**	**31.41**	**30.72**	**31.54**	**32.56**	**39.03**	**35.64**	**35.04**	**33.83**	**34.51**	**33.61**	**37.08**	**35.38**	**34.04**	**32.70**	**32.07**	**30.92**
			**Oxygenated monoterpenes**	**62.77**	**65.59**	**64.34**	**64.61**	**64.68**	**64.28**	**55.41**	**58.96**	**61.16**	**62.17**	**61.20**	**61.95**	**54.73**	**58.10**	**60.91**	**63.83**	**64.05**	**65.47**
			**Sesquiterpenes**	**4.52**	**3.50**	**3.62**	**3.80**	**3.20**	**2.70**	**5.05**	**5.06**	**3.50**	**3.63**	**3.83**	**3.67**	**5.70**	**5.81**	**4.59**	**3.16**	**3.49**	**3.44**
			**Others**	**0.17**	**0.07**	**0.06**	**0.09**	**0.04**	**0.04**	**0.10**	**0.05**	**0.04**	**0.03**	**0.05**	**0.03**	**0.10**	**0.04**	**0.03**	**0.04**	**0.04**	**0.02**

^a^ The numbering refers to elution order, and values (relative peak area percent) represent averages of 3 determinations; ^b^ retention index (RI) relative to standard mixture of *n*-alkanes on SPB-5 column. ^c^ Literature retention index (RI) [42].

**Table 4:** Correlation coefficients and *p* values (in Italic) among the major compounds of the EO of *Origanum heracleoticum* cultivated in a 3-year trial in 3 Sicilian localities; *n* = 54. Thy: thymol; carv: carvacrol; *p*-cym: *p*-cymene; γ-terp: γ-terpinene.

	**Thy**	**Carv**	***p*-cym**	**γ-te** **rp**	**Thy+carv**	***p*-cym + γ-terp**	**Thy+carv + *p*-cym + γ-terp**	**TS (°C)**	**Rainfall (mm)**
**Thy**	-	0.323 *(0.017)*	−0.673 *(<0.001)*	−0.816 *(<0.001)*	0.999 *(<0.001)*	−0.882 *(<0.001)*	0.826 *(<0.001)*	0.049 *(0.725)*	−0.340 *(0.012)*
**Carv**		-	−0.013 *(0.926)*	−0.160 *(0.247)*	0.349 *(0.010)*	−0.126 *(0.363)*	0.498 *(<0.001)*	0.182 *(0.189)*	−0.653 *(<0.001)*
***p*-cym**			-	0.457 *(0.001)*	−0.666 *(<0.001)*	0.740 *(<0.001)*	−0.375 *(0.005)*	0.287 *(0.035)*	0.179 *(0.196)*
**γ-terp**				-	−0.813 *(<0.001)*	0.936 *(<0.001)*	−0.418 *(0.002)*	0.025 *(0.858)*	0.110 *(0.428)*
**Thy+carv**					-	−0.877 *(<0.001)*	0.832 *(<0.001)*	0.054 *(0.700)*	−0.356 *(0.008)*
***p*-cym + γ-terp**						-	−0.464 *(<0.001)*	0.132 *(0.340)*	0.154 *(0.267)*
**Thy + carv + *p*-cym + γ-terp**							-	0.252 *(0.066)*	−0.479 *(<0.001*)

**Table 5:** *O. heracleoticum*. Results of ANOVA (*F* values and significance level) for the yields and the major components of the EOs obtained throughout the trial.

	Source of Variability					
	Year (Y)	Location (L)	Y × L	Survey (Location)	Error	Total
**DF**	2	2	4	15	30	53
**Oil Yield (% *v/w*)**	14.79 *** ^1^	1.25 n.s.	<1 n.s.	4.13 ***		
**Monoterpene hydrocarbons (MH)**						
α-thujene	38.45 ***	<1 n.s.	<1 n.s.	2.51 *		
α-pinene	47.19 ***	<1 n.s.	<1 n.s.	2.85 **		
camphene	28.15 ***	<1 n.s.	<1 n.s.	3.83 ***		
sabinene	3.06 n.s.	10.09 **	3.05 *	<1 n.s.		
β-pinene	31.71 ***	<1 n.s.	1.80 n.s.	3.76 ***		
myrcene	27.79 ***	<1 n.s.	<1 n.s.	2.86 **		
α-phellandrene	13.94 ***	<1 n.s.	<1 n.s.	2.68 **		
α-terpinene	7.60 **	<1 n.s.	<1 n.s.	1.54 n.s.		
*p*-cymene	25.57 ***	<1 n.s.	<1 n.s.	1.86 n.s.		
limonene	23.00 ***	<1 n.s.	<1 n.s.	2.85 **		
*cis*-ocimene	14.98 ***	<1 n.s.	<1 n.s.	1.12 n.s.		
*trans*-ocimene	24.13 ***	13.77 ***	9.27 ***	2.72 **		
γ-terpinene	6.83 **	4.34*	<1 n.s.	<1 n.s.		
terpinolene	13.91 ***	<1 n.s.	1.63 n.s.	2.33 *		
Other MH	42.46 ***	1.97 n.s.	2.38 n.s.	1.78 n.s.		
**Total MH**	16.85 ***	1.37 n.s.	<1 n.s.	1.13 n.s.		
**Oxygenated monoterpenes (OM)**						
*cis*-sabinene hydrate	20.84 ***	<1 n.s.	<1 n.s.	2.87 **		
linalool	4.67 *	<1 n.s.	1.05 n.s.	1.85 n.s.		
borneol	25.78 ***	1.07 n.s.	2.85 *	1.25 n.s.		
terpinen-4-ol	6.55 **	<1 n.s.	2.11 n.s.	<1 n.s.		
α-terpineol	4.20 *	3.32 n.s.	<1 n.s.	<1 n.s.		
thymol methyl ether	6.78 **	<1 n.s.	<1 n.s.	1.73 n.s.		
carvacrol methyl ether	11.06 ***	<1 n.s.	1.86 n.s.	6.46 ***		
thymol	18.88 ***	<1 n.s.	<1 n.s.	1.16 n.s.		
carvacrol	73.10 ***	1.96 n.s.	13.20 ***	1.19 n.s.		
Other OM	33.34 ***	1.13 n.s.	3.14 *	3.00 **		
**Total OM**	17.52 ***	<1 n.s.	<1 n.s.	1.20 n.s.		
**Total monoterpenes (MH + OM)**	53.94 ***	<1 n.s.	1.03 n.s.	2.42 *		
**Sesquiterpenes (ST)**						
β-caryophyllene	59.54 ***	<1 n.s.	1.30 n.s.	1.30 n.s.		
α-humulene	60.8 ***	<1 n.s.	<1 n.s.	<1 n.s.		
germacrene D	44.67 ***	3.15 n.s.	<1 n.s.	<1 n.s.		
β-bisabolene	74.08 ***	<1 n.s.	<1 n.s.	1.51 n.s.		
δ-cadinene	9.85 ***	<1 n.s.	<1 n.s.	<1 n.s.		
spathulenol	7.77 **	1.31 n.s.	1.41 n.s.	<1 n.s.		
**Total ST**	66.53 ***	<1 n.s.	<1 n.s.	1.71 n.s.		
*p*-cymene + γ-terpinene	15.54 ***	2.01 n.s.	<1 n.s.	<1 n.s.		
thymol + carvacrol	19.52 ***	<1 n.s.	<1 n.s.	1.18 n.s.		
*p*-cymene + γ-terpinene + thymol + carvacrol	19.87 ***	<1 n.s.	<1 n.s.	1.11 n.s.		

^1^ Significance levels: ***: *p* ≤ 0.001; **: 0.001< *p* ≤0.01; *: 0.01 < *p* ≤0.05; n.s. *p* > 0.05 (not significant).
